# Dynamics of influenza A virus infections in permanently infected pig farms: evidence of recurrent infections, circulation of several swine influenza viruses and reassortment events

**DOI:** 10.1186/1297-9716-44-72

**Published:** 2013-09-04

**Authors:** Nicolas Rose, Séverine Hervé, Eric Eveno, Nicolas Barbier, Florent Eono, Virginie Dorenlor, Mathieu Andraud, Claire Camsusou, François Madec, Gaëlle Simon

**Affiliations:** 1 Anses, Laboratoire de Ploufragan/Plouzané, Unité Epidémiologie et Bien-Être du Porc, BP 53, 22440 Ploufragan, France; 2 Anses, Laboratoire de Ploufragan/Plouzané, Unité Virologie Immunologie Porcines, BP 53, 22440 Ploufragan, France; 3 Université Européenne de Bretagne, Rennes, France

## Abstract

Concomitant infections by different influenza A virus subtypes within pig farms increase the risk of new reassortant virus emergence. The aims of this study were to characterize the epidemiology of recurrent swine influenza virus infections and identify their main determinants. A follow-up study was carried out in 3 selected farms known to be affected by repeated influenza infections. Three batches of pigs were followed within each farm from birth to slaughter through a representative sample of 40 piglets per batch. Piglets were monitored individually on a monthly basis for serology and clinical parameters. When a flu outbreak occurred, daily virological and clinical investigations were carried out for two weeks. Influenza outbreaks, confirmed by influenza A virus detection, were reported at least once in each batch. These outbreaks occurred at a constant age within farms and were correlated with an increased frequency of sneezing and coughing fits. H1N1 and H1N2 viruses from European enzootic subtypes and reassortants between viruses from these lineages were consecutively and sometimes simultaneously identified depending on the batch, suggesting virus co-circulations at the farm, batch and sometimes individual levels. The estimated reproduction ratio *R* of influenza outbreaks ranged between 2.5 [1.9-2.9] and 6.9 [4.1-10.5] according to the age at infection-time and serological status of infected piglets. Duration of shedding was influenced by the age at infection time, the serological status of the dam and mingling practices. An impaired humoral response was identified in piglets infected at a time when they still presented maternally-derived antibodies.

## Introduction

Swine flu is mainly caused by influenza type A viruses and several subtypes of swine influenza viruses (SIVs) have become enzootic in the pig population. Indeed, three H1N1, H1N2 and H3N2 SIVs, are currently circulating among pigs worldwide, and owing to various mechanisms of emergence, genetic lineages may vary within each subtype depending on the geographical location (North America, Europe and Asia) [1,2]. Viruses from the European avian-like swine H1N1 (H1_av_N1) and the human-like reassortant swine H1N2 (H1_hu_N2) lineages, as well as viruses originating from reassortment between these two enzootic SIVs are the main strains detected in the French pig population [3,4]. These viruses are responsible for a respiratory syndrome similar to human flu, including pyrexia, anorexia, lethargy, cough and often growth retardation [1,5]. Swine influenza is well known to farmers and veterinarians and often has been described as an occasional outbreak with a time-limited impact on herd health in a context of scarce bacterial complications. However, recent findings have shown that SIVs particularly those of the H1_av_N1 subtype, are major co-factors of Porcine Respiratory Disease Complex (PRDC) and significantly increase the severity of respiratory diseases under experimental [6] or farm conditions [7]. Swine flu is generally an epizootic infection spreading rapidly within the herds and fading out within two weeks or so [1]. However, as early as the 1980’s some authors reported the ability of SIVs to persist within farrow-to-finish farms between two outbreaks [8]. The serological follow-up of sentinel farms in 4 different European countries for 3 years showed that some farms tested positive for one specific subtype in all sampling periods, suggesting possible virus persistence on the farm [9]. This enzootic within-farm persistence of SIVs has recently been described as consecutive waves of diverse intensity in some Spanish farrow-to-finish operations [10]. Recurrent swine flu has been more and more frequently reported by swine practitioners. In 2011, 30% of the influenza outbreaks reported by the French national surveillance network for SIVs were described as recurrent infections [4]. They generally occur in nursery and can affect all the batches at a particular age and are responsible for a permanent destabilization of herd health with respiratory or sometimes digestive complications. The Spanish study highlighted the possible co-circulation of different subtypes or different variants of a given subtype in the same batch of pigs [10]. These co-circulation events increase the probability of reassortments, possibly leading to the emergence of new viruses more pathogenic for pigs and with severe outcomes, as reported in French pig herds in 1984 following the introduction of a new H3N2 subtype [11]. Moreover, the risk of generation of novel SIVs that can be transmitted to humans and have the ability to further spread within human populations has also to be considered as swine flu is recognized as a zoonosis [2]. In 2009, emergence in humans of a pandemic H1N1 (H1N1pdm) virus that contains gene segments with ancestors in North American and Eurasian SIV lineages reminded this risk [12]. Since then, H1N1pdm entered the pig population and reassortment events with different enzootic SIVs have been then reported worldwide [13-17], one of them having being responsible of many human infections in the US [18-20].

The characteristics of these recurrent SIV infections are poorly known. The conditions leading to these recurrent infections are not well understood and the consequences of these repeated infections in terms of emergence of new reassortant viruses and herd immunity have not been described to date. The objectives of this study were (i) to identify viruses involved in these recurrent SIV infections, (ii) to estimate the quantitative parameters characterizing the dynamics of infection and (iii) to identify the main characteristics possibly involved in the recurrence mechanism. This study was designed as a cohort study and was carried out in farrow-to-finish pig farms naturally affected by recurrent SIV infections.

## Materials and methods

### Ethical statement

This study was carried out in strict accordance with the guidelines of the Good Experimental Practices (GEP) standard adopted by the European Union. All experimental procedures were conducted in accordance with the recommendations given by the Anses / ENVA/UPEC ethical committee (agreement #16 to the National committee for ethics in animal experimentation). The study was conducted under the responsibility of a main investigator (NR) who has an individual agreement for animal experimentation (agreement #B22030).

### Selection of target farms for the cohort study

Candidate farms (*n* = 10) were proposed by veterinarians involved in swine operations, according to their knowledge of presumed recurrent influenza outbreaks. To confirm the SIV etiology of the reported recurrent respiratory syndromes, nasal swabs (MW950(S) Virocult®, KITVIA, Labarthe-Inard, France) were taken from 10 pigs with pyrexia (> 40.5 °C rectal temperature) during a clinical outbreak representative of the recurrent respiratory outbreaks observed in the farm. Paired blood samples were taken from each of the selected pigs, one at the time of the outbreak and the other 21 days later. Three farms, #A, #B and #C, located in Brittany France were confirmed as SIV positive by M gene RT-PCR (see below) at the time of the outbreak, and were retained for the detailed follow-up study. They had 150, 350 and 770 sows divided into 5, 10 and 20 batches respectively, with 28, 30 and 36 sows, respectively, per batch. According to the batch-rearing system, the time-interval between 2 batches was 4, 2 and 1 weeks for Farm#A, #B and #C, respectively.

### Follow-up study in selected farms

The three farms (#A, #B and #C) were subjected to the same protocol based on the individual follow-up of a cohort of piglets from birth to slaughter. The follow-up was repeated on 3 consecutive batches in each farm except in Farm#C for which every other batch was considered because of the small in-between interval of 1 week. The follow-up lasted from 7 to 9 months in each farm and started in January 2011, ending with the last animal slaughtered in April 2012. A representative sample of 40 piglets per batch was constituted at birth. All piglets were identified in every litter and 4 piglets per litter were randomly selected from 10 sows which were randomly selected from sows due to farrow in the considered batch. The randomization of sow selection, took into account sow parity through a stratification process (gilts, parities 1–2, 3–4 and 5 or more). Randomly selected piglets to be individually monitored throughout the follow-up period were identified (tattoo and ear-tag) and kept with their native dam. Cross-fostering was allowed for the other littermates. The monitored piglets were then reared with other piglets in the batch and subjected to the same practices as other piglets in the farm. The sows in all 3 farms were vaccinated with the commercial trivalent (H1N1, H1N2 and H3N2) vaccine GRIPOVAC 3® (Merial, Lyon, France) according to the same protocol, i.e. primo-vaccination of gilts involving 2 injections 3 weeks apart and a booster injection before farrowing. Thus 360 piglets, in total, were individually monitored in this study.

### Sampling procedure and clinical examinations

Blood samples were taken from monitored piglets at 1, 6, 10, 14, 18, and 22 weeks of age and at slaughter. Blood samples were also taken from the related dams one week after farrowing to assess the transfer of maternal antibodies to the piglets through colostrum. Samples were collected by jugular vein puncture, using evacuated tubes (Vacuette, Dutscher SAS, Brumath, France) without additive. Sera were obtained by centrifugation for 10 min at 3500 × *g* and stored at −20 °C until subsequent analysis. Clinical observations including coughs, coughing fits and sneezing frequency were evaluated at each sampling date (3 consecutive counts of 2 min each to calculate the relative number of coughs/100 animals).

When a respiratory outbreak was detected by the farmer, nasal swabs were taken from the monitored piglets each day for the 5 first days at least and then every 2 days the following week to assess the evolution of the frequency of SIV shedding piglets over time. At each sampling time, the rectal temperature of individual piglets was recorded and cough, coughing fits and sneezing frequency were estimated at the group level. Two additional blood samples were taken at the beginning of the outbreak (early sample) and 21 days later (late sample), respectively. Nasal swabs were immediately stored at + 4 °C for transport and further frozen at −70 °C until virological analysis.

The carcasses of followed animals were examined at slaughter. Lungs were removed from the slaughter-line for individual macroscopic examination, palpated and visually appraised for pneumonia-like gross lesions and pleuritis according to the method described by Madec and Kobisch [21]. Pneumonia gross lesions consisted of dark red to greyish purple areas of consolidation in the cranial, middle, accessory and/or caudal lobes. Pneumonia-like gross lesions were scored from 0 to 4 on each of the seven lobes, which gave a maximum possible score of 28 if the entire lung was affected. Pleuritis lesions, i.e., inflammation of the visceral and parietal pleura, were graded from 0 (no lesion) to 4 (adherence of the entire lung to the rib cage).

### Sample analyses

#### Detection of influenza A virus genome by RT-PCR

Influenza A virus genome was detected in nasal swab supernatants by M gene real-time RT-PCR using the TaqVet™ Swine Influenza A - A/H1N1/2009 included Kit (Laboratoire Service International, Lissieu, France). This commercial assay had been previously validated by the French National Reference Laboratory for Swine Influenza [22] and was used according to the manufacturer’s instructions.

Results are interpreted according to cycle threshold (Ct) values obtained for each sample, i.e., genome detected (Ct < 45) or not detected (No Ct). Although this method is qualitative, it is generally accepted that for samples of the same type and analysed simultaneously, the lower the Ct value, the higher the viral genome load in the sample. Because it has been shown that virus isolation in cell culture is generally unsuccessful when Ct values of samples are between 35 and 45 (unpublished results), it was hypothesized in this study that piglets would only shed enough viral particles in their nasal fluid to infect other animals when Ct values were below 35.

#### Virus characterization by molecular subtyping

Influenza A viruses detected in nasal swab supernatants were identified by subsequent RT-PCR assays designed to specifically amplify HA or NA genes belonging to the SIVs in circulation in the European pig population, i.e. H1_av_N1, H1_hu_N2, H3N2 and H1N1pdm viruses. Thus, M gene positive RNA extracts were first subjected to real-time RT-PCR assays targeting H1 or N1 genes of the H1N1pdm virus, using the “TaqVet™ Swine Influenza A/H1N1 2009 – H1 detection” kit and/or the “TaqVet™ Swine Influenza A/H1N1 2009 – N1 detection” kit (Laboratoire Service International, Lissieu, France), respectively [22]. Then, two conventional multiplex RT-PCR assays were carried out on M gene positive RNA extracts with Ct values below 35, according to the methods proposed by Chiapponi et al. [23]. One multiplex RT-PCR assay allows the specific detection of haemagglutinin genes of H1_av_, H1_hu_ and H3 lineages, while the other assay permits the amplification of neuraminidase genes of N1 and N2 lineages. In case analyses of the biological sample were unsuccessful, virus isolation was attempted in Madin Darbin Kidney Canine (MDCK) cell cultures and molecular sub-typing was renewed on the amplified viral RNA. When 35 < Ct < 45, the quantity of virus present was too low for direct subtyping by conventional multiplex RT-PCRs or virus isolation and thus, further identification.

#### Detection of SIV antibodies by haemagglutination inhibition test

Antibodies against European subtypes H1_av_N1, H1_hu_N2 and H3N2 were detected and titrated using haemagglutination inhibition (HI) tests in sera collected at a fixed age as well as in early and late blood samples taken at the time of a respiratory outbreak. HI tests were performed according to standard procedures [24]. Non-specific inhibitors of haemagglutination and agglutination factors were removed by treatment of the sera with receptor-destroying enzyme (RDE) and adsorption onto chicken erythrocytes. Two-fold serum dilutions were tested starting at a dilution of 1:10. Virus strains A/Swine/Cotes d’Armor/0388/09 (H1_av_N1), A/Swine/Scotland/410440/94 (H1_hu_N2) and A/Swine/Flandres/1/98 (H3N2) were used as reference antigens provided by the European Surveillance Network for Influenza in Pigs [25]. HI tests were performed using 4 haemagglutinating units (HAU) of virus and 0.5% chicken red blood cells. Titres were expressed as the reciprocal of the highest dilution inhibiting 4 HAU [26], and were subjected to log2 transformation for statistical analysis and graphical representation. Some selected sera were also analyzed by HI tests using viruses isolated on-farm after virus amplification on cell-cultures (homologous HI tests) and by ID Screen® Antibody Influenza A Competition ELISA kit (IDVet, Montpellier, France) for the detection of anti-nucleoprotein antibodies.

#### Serological analyses for other respiratory pathogens

Early and late blood samples from each outbreak were analyzed to detect the likelihood of another infection simultaneous to SIV. Thus, antibodies directed towards *Myco-plasma hyopneumoniae* (ELISA test, OXOID, Basingstoke, RU), Porcine Reproductive and Respiratory Syndrome virus (PRRSV) (ELISA HerdCheck PRRS X3, IDDEX, Hoofddorp, Pays-Bas) and Porcine Circovirus type 2 (PCV-2) [27] were tested in these sera samples.

### Statistical analyses

#### Factors associated with early SIV shedding and seroconversion

The age at first viral shedding and the age at seroconversion were examined from a survival analysis. This analysis was aimed to identify the piglet characteristics associated with (i) initiation of the infectious process and (ii) seroconversion following infection, respectively. A multivariable Cox proportional hazard regression model was used to relate variables to both outcomes [28]. The candidate variables tested as regards time to first shedding were: gender of the piglet, mean HI titre (3 subtypes) of the dam, mean HI titre of the piglet (1 week of age), number of cross-fostered piglets in the litter, number of stillborn and mummified piglets in the litter, sow parity. The variables tested as regards time to seroconversion were: gender of the piglet, subtype-specific HI titre of the dam, subtype-specific HI titre of the piglet (1 week of age), number of cross-fostered piglets in the litter, number of stillborn and mummified piglets in the litter, sow parity, and age at infection. Only variables associated with the outcome (*p* < 0.20) in a preliminary univariate selection were included in a full multivariable model. Correlations between candidate variables were also tested to prevent from multicollinearity in the multivariate analysis. A backward selection was then applied to only select those variables significantly related to the outcome (*p* < 0.05) in the final model.

#### Quantification of SIV outbreak dynamics through R estimation

The intensity of SIV spread within the population was determined by estimating the reproduction ratio (*R*). We used the method of exponential growth of the epidemic [29] based on the cumulated incidence data obtained in each viral outbreak. The exponential growth (*r*) and its confidence interval were estimated from a Poisson regression [30,31] on the daily cumulated incidence of cases over the time period when the increase of incident cases could be considered as exponential. This estimation was only possible for those outbreaks in which the number of days of sampling during the growing phase of the epidemic was sufficient (at least 3). Some outbreak data could not be used because almost all the piglets were already shedding virus at the first sampling date. The underlying infectious process was deemed to follow a SEIR (Susceptible-Exposed-Infectious-Recovered) class of epidemic models. From this classical model, the latent and infectious periods are exponentially distributed with rates *b*_*1*_ and *b*_*2*_, respectively. In consequence, the generation interval distribution is implicitly the convolution of two exponential distributions with mean Tc=1b1+1b2. To estimate *R*, we therefore used M gene RT-PCR data to determine when piglets were infected but were unlikely to transmit the virus (latent period) and when they shed enough virus particles for transmission to susceptible animals (infectious period). The evolution of Ct values with time was modelled for each pig by a polynomial regression (2^nd^ order) and the corresponding equation was solved to find solutions corresponding to Ct = 35 and Ct = 45. The latent period L=1b1 was determined as the time-period when 35 < Ct < 45 and the infectious period I=1b2 corresponded to the time interval when Ct values remained below 35 (Additional file 1). Only polynomial regressions for which the adjusted R^2^ was above 0.80 were used to estimate *L* and *I* duration. The average values for all piglets in a given outbreak were incorporated in the *R* estimation according to the equation R=1+rb11+rb2[29].

The characteristics of piglets associated with the duration of latency or infectiousness were assessed by ANOVA. All statistical analyses were done using the software R 3.0.0 [32].

## Results

### Description of influenza outbreaks and confirmation of their etiology

#### Clinical parameters

**Figure 1:**
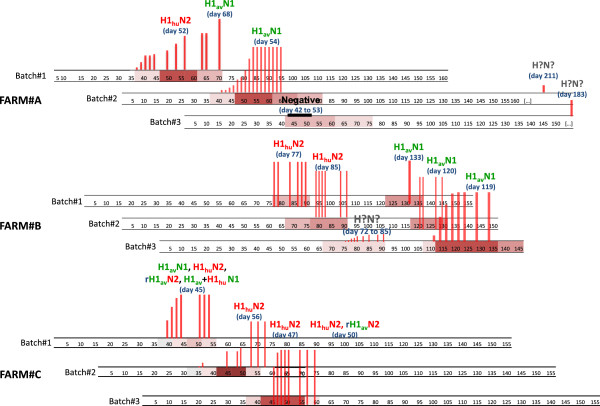
**Description of influenza-like outbreaks observed in the monitored piglets (3 farms, 3 batches per farm).** Representation of clinical outbreaks and clinical severity on each batch-specific age time scale. Clinical severity: from mild “pink box symbol” to acute “maroon box symbol”. Red vertical bars correspond to the cumulated incidence of SIV positive pigs, the maximum size bar representing the 40 monitored piglets. SIV subtypes are indicated on each SIV outbreak with the age corresponding to the virus identification.

**Figure 2:**
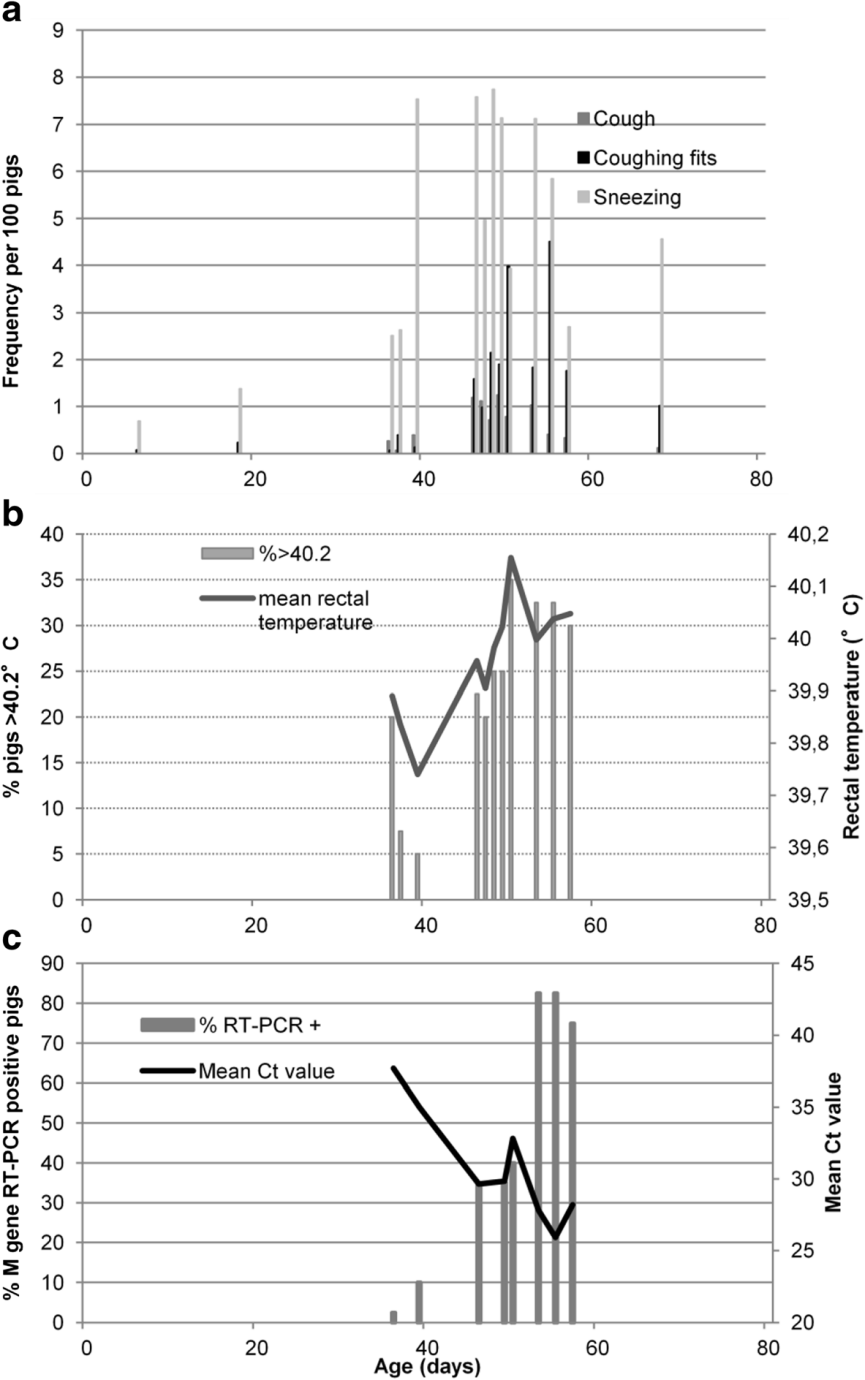
**Correspondence between clinical (cough, sneezing, rectal temperature) and virological data in a typical influenza outbreak (Farm#C, batch#2, nursery period). (a)**: frequency of sneezing, cough and coughing fits for 100 pigs (mean value of 3 counts, 2 minutes each). **(b)**: mean rectal temperature of the 40 monitored piglets (solid grey line) and frequency of piglets above 40.2 °C (grey bars). **(c)**: percentage of M gene RT-PCR positive piglets (grey bars) and mean Ct value (solid grey line).

Respiratory influenza-like outbreaks were observed in every followed batch in all 3 farms, with even 2 consecutive outbreaks in two batches from Farm#A and in every batch from Farm#B (Figure 1). Piglets were affected in nursery in farms #A and #C, from 40 days old on average, with peak clinical manifestations observed around 50 days of age. In Farm#B, the first outbreak occurred at the beginning of the fattening period between 70 and 90 days of age and the second one when the pigs were about 120 days old. Considerable within farm repeatability was observed, the piglets from successive batches being systematically affected at the same period. The intensity of severity of influenza outbreaks varied according to the farm and between batches in the same farm. Piglets affected in nursery (farms #A and #C) were mainly characterized by pyrexia (40 °C and more), a high frequency of sneezing, coughs and coughing fits, the frequency of these latter increasing considerably 5 to 10 days after the first clinical signs (Figure 2). When piglets were affected during the fattening phase (Farm#B), the symptoms were globally more severe than in nursery especially for the second outbreak in batch#3. These animals were characterized by severe lethargy and anorexia, leading to considerable growth retardation as attested by the carcass weight at slaughter age (Table 1).

**Table 1:** Respiratory lesions and slaughter characteristics of followed pigs (3 farms, 3 batches/farm).

	**Farm#A**			**Farm#B**			**Farm#C**		
	**Batch#1**	**Batch#2**	**Batch#3**	**Batch#1**	**Batch#2**	**Batch#3**	**Batch#1**	**Batch#2**	**Batch#3**
Pneumonia (%)	52.8	39.5	61.9	14.3	25.8	73.0	9.4	10.5	15.8
Pneumonia mark/28 (sd)	2.9 (3.9)	1.5 (3.4)	3.6 (5.6)	0.3 (0.9)	1.9 (4.0)	3.8 (4.7)	0.2 (0.6)	0.2 (0.5)	0.3 (0.9)
Pleuritis (% marks >2)	2.9	0	0	0	0	5.3	3.0	0	0
Pneumonia healings (%)	11.1	2.6	4.8	4.8	0	7.9	6.3	13.2	21.1
Abscesses (%)	0	2.6	9.5	0	0	5.6	0	2.6	0
Nodule (%)	0	2.6	0	0	0	0	0	0	0
Edema (%)	2.8	0	14.3	0	6.5	7.9	0	5.3	0
Trach.-bronch. lymph nodes									
Congestion (%)	8.3	2.6	42.9	0	12.9	10.5	3.1	0	5.3
Hypertrophy (%)	13.9	10.5	23.8	0	12.9	13.2	3.1	2.6	2.6
Number of observed pigs	36	38	21	21	31	38	32	38	38
Carcass weight in kg (sd)	93.5 (2.8)	92.2 (3.0)	91.6 (2.5)	85.3 (7.0)	86.9 (2.7)	89.4 (7.6)	94.5 (6.0)	92.5 (7.5)	94.3 (4.1)
Slaughter age in days (sd)	181.3 (8.4)	176.9 (13.5)	177.7 (13.0)	183.4 (14.1)	180.6 (10.3)	185.2 (9.7)	174.8 (7.2)	171.8 (8.0)	169.7 (7.5)

Characteristics at slaughter were moderately affected except for pigs from Farm#B where the piglets had been infected during the fattening phase (Table 1). However, a large proportion of pigs exhibited pneumonia lesions, which were relatively moderate in Farm#A (batches #1 and #2), and more severe in Farm#B, batch#3 (Table 1). Severe pleuritis lesions were observed in 5.3% of pigs in the same batch. A high frequency of pneumonia associated with interlobular edema (14.3%) was observed in pigs from Farm#A, batch#3 which had been detected as SIV-infected shortly before shipment to the slaughterhouse. A high proportion of pigs in farms #A and #C displayed signs of pneumonia healing related to early infections.

#### Virological results

All but one clinical outbreak were confirmed as related to SIV etiology (Figure 1). In the first outbreak on Farm#A, batch#3, all M gene RT-PCRs remained negative for the 40 piglets at all sampling times. In the same farm, a late SIV infection was detected when the animals were due to leave for the slaughterhouse (batches #2 and #3). The cumulated incidence of M gene positive piglets generally increased less rapidly when SIV outbreaks occurred in nursery (farms #A and #C) than during fattening (Farm#B). All piglets were found positive at the first sampling date for all outbreaks of Farm#B but one (batch #3) (Figure 1).

#### Relation between clinical and virological parameters

An outbreak (Farm#C, batch#2) with detailed clinical and virological results was taken as an example of observed SIV outbreaks occurring in nursery (Figure 2). Comparison of the clinical parameters (coughs, sneezing and rectal temperature) and virological data showed a prodromal phase with an increase of sneezing frequency, a small (≤ 20%) proportion of animals with pyrexia and some piglets detected as SIV positive but with low shedding (high Ct values). In the state phase, sneezing was associated with an increased frequency of cough and coughing fits (Figure 2a), a high (> 20%) proportion of pigs with pyrexia (> 40.2 °C) and a general increase in the group-level average rectal temperature (Figure 2b). The proportion of SIV positive pigs then increased considerably and was associated with high virus shedding (low Ct values, Figure 2c).

### Identification of influenza A viruses responsible for the outbreaks

Detected viruses could be characterized for all confirmed SIV outbreaks, except the first infection detected in batch#3 of Farm#B, owing to the low frequency of infected pigs and the limited amount of virus material in the samples (low shedding). Viruses of H1_av_N1 and H1_hu_N2 subtypes were successively identified in each of the 3 studied farms (Figure 1). Both virus subtypes were also detected within a same batch, either from 2 consecutive and distinct outbreaks (Farm#B) or during the same global outbreak (Farm#A, batch#1 and Farm#C, batch#1), and even from the same animal (Farm#C, batch#1). In addition, an atypical virus of rH1_av_N2 subtype was also detected in this farm, confirming the occurrence of reassortment events due to the co-circulation of both enzootic lineages at the same time in the same batch (Figure 1). Viruses of H3N2 and H1N1pdm lineages were not detected in this study.

### Serological profiles against SIVs

#### Sows

**Figure 3:**
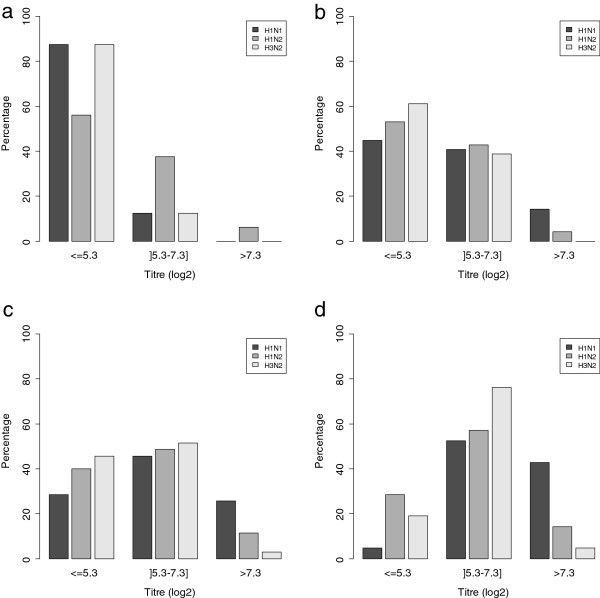
**Distribution of the percentage of sows according to their serological HI titre.** HI serological titres (log2 transformed) of sera taken from sows one week post farrowing as regards subtypes H1_av_N1, H1_hu_N2 and H3N2 and for different parity groups: gilts **(a)**, parity 1–2 **(b)**, parity 3–4 **(c)** and parity 5 and more **(d)**.

The sows on all three farms were vaccinated against the 3 virus subtypes, H1_av_N1, H1_hu_N2 and H3N2. Thus, distribution of dam serological titres at 1 week post-farrowing was influenced by parity because of booster vaccine injection at each reproductive cycle (Figure 3). Concerning H3N2, which was not circulating in the three investigated farms, sows had mainly low specific HI titres until the second pregnancy (Figure 3a), whereas the majority of older sows had moderate titres below 160 (log2(titre) < 7.3) (Figure 3d). The same global evolution was observed for the presence of antibodies against H1_av_N1 and H1_hu_N2, although high serological titres (> 160, log2(titre) > 7.3) were observed in a non negligible proportion of sows beyond parity 3, suggesting permanent exposure of the reproductive herd in these farms to H1_av_N1 and H1_hu_N2 infections despite vaccination (Figure 3c, d).

#### Growing pigs

**Figure 4:**
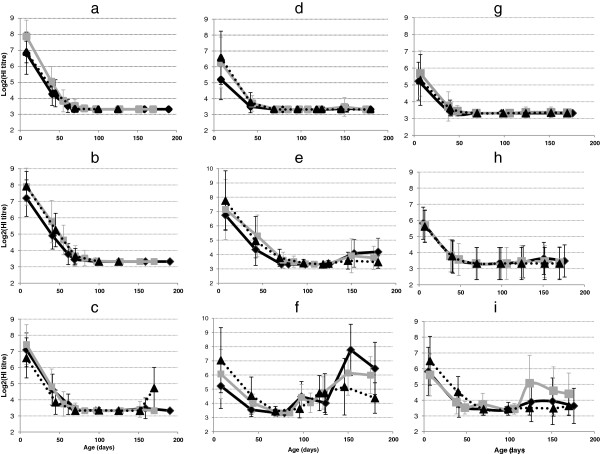
**Serological (HI tests) titres (log2 transformed) of monitored piglets.** Farms are in columns: Farm#A **(a,b,c)**, Farm#B **(d,e,f)** and Farm#C **(g,h,i)**. SIV subtypes are in rows: H3N2 (row a-d-g for farms #A, #B and #C respectively), H1N1 (row b-e-h for farms #A, #B and #C respectively) and H1N2 (row c-f-i for farms #A, #B and #C respectively). In each graph, 3 batches are represented: batch#1: dark solid line, batch#2: grey solid line, batch#3: dark dotted line. Results from age-fixed visits as well as supplementary samples related to influenza outbreaks are displayed.

Because of sow vaccination, all piglets tested positive at 1 week of age for antibodies against the three subtypes H1_av_N1, H1_hu_N2 and H3N2, in close agreement with the serological status of the dams one week after farrowing (Figure 3 and Figure 4). The serological results for H1_av_N1, H1_hu_N2 and H3N2 between batches from a given farm were relatively homogeneous. The HI titres corresponding to the three subtypes then decreased in relation to the diminution of maternal antibodies with time until 70 days in farms #A and #B and 50 days in Farm#C (Figure 4). No specific seroconversion was observed for the H3N2 subtype (Figures 4a, d, g), in agreement with the absence of isolation of this virus strain. In Farm#A, no seroconversion was detected in pigs from the first 2 batches, whereas H1_av_N1 and H1_hu_N2 virus infections were confirmed (Figures 4b, c). A late seroconversion towards H1_hu_N2 was observed in batch#3, in agreement with the late detection of SIV in these animals and suggesting the identity of the virus involved. In Farm#B, a first seroconversion towards H1_hu_N2 subtype was observed after 90 days of age in batches #1 and #2, in agreement with the identification of H1_hu_N2 viruses at the time of outbreaks occurring from 70 days of age. A second increase in H1_hu_N2 antibodies titres was further observed after 120 days of age although the virus responsible for the second outbreak during fattening belonged to the H1_av_N1 lineage (Figure 4f). A slight increase in H1_av_N1 HI titres was observed later, but without marked seroconversion. In Farm#C, no specific seroconversion of either H1_hu_N2 or H1_av_N1 was observed, although there was evidence of systematic co-circulation of both subtypes as well as reassortant in all but one batch. Only a highly delayed seroconversion to the H1_hu_N2 subtype at 120 days of age was observed in batch#2 (Figure 4i), but its linkage to the outbreak detected at 50 days of age was unlikely. This seroconversion might be related to an asymptomatic SIV infection occurring during fattening.

### Seroconversion as regards other respiratory pathogens

No seroconversion for *Mycoplasma hyopneumoniae* or PCV-2 was detected at the time of influenza outbreaks in farms #A and #C. A specific PRRSV seroconversion was only detected concomitantly to the second SIV outbreak in Farm#B, batch#3 (data not shown).

### Quantification of SIV outbreak dynamics through R estimation

*R* estimates could be calculated for Farm#A batches #1 and #2, Farm#B batch #3 and Farm#C batches #1 and #2 as a sufficient number of early samples was obtained at the beginning of the outbreak to estimate the growth rate of the epidemic (Figure 1). The *R* estimates varied between 2.5 [_95% CI_ 1.9-2.9] and 6.9 [_95% CI_ 4.1 – 10.5] according to the farms and batches (Table 2). *R* estimate was largest for the outbreak detected in Farm#B batch #3 when the pigs were 120 days old. This large *R* value was mainly due to a significantly higher growth rate of the epidemic (*r*) as compared to the other outbreaks investigated. The estimated duration of infectiousness was between 6.0 and 10.4 days, leading to large *R* estimates in some outbreaks occurring in young piglets with small *r* values (Farm#C, batch #2). There was no apparent relationship between the estimated parameters and the virus subtype or the diversity of viruses identified during a single outbreak. However, the duration of infectiousness was significantly shorter when piglets were born to dams delivering high titres of SIV maternal antibodies and dams with parity > 4 (Table 3). Both latency and infectiousness were of longer duration in piglets infected before 50 days of age. Latency was also longer in piglets born to sows that received a large number of cross-fostered piglets (> 4) (Table 3).

**Table 2:** **Reproduction ratio (*R*), duration of latency and infectiousness estimations for different influenza outbreaks.**

**Farm**	**Batch**	**Age period (days) at SIV infection**	**SIV subtypes**	**Exponential growth rate (r) [95% CI]**	**Latency**^**a**^**in days (sd)**	**Infectiousness**^**b**^**in days (sd)**	***R***^**c**^**[95% CI]**
A	1	39-56	H1_hu_N2, H1_av_N1	0.15 [0.10 – 0.19]	2.2 (1.0)	5.6 (2.6)	2.5 [1.91-2.94]
	2	38-64	H1_av_N1	0.18 [0.14 – 0.21]	2.2 (0.87)	7.5 (2.4)	3.2 [2.72-3.82]
B	3	106-127	H1_av_N1	0.52 [0.31-0.72]	1.4 (0.42)	6.0 (1.5)	6.9 [4.12-10.50]
C	1	42-50	H1_av_N1, H1_hu_N2, rH1_av_N2	0.26 [0.10-0.43]	1.4 (0.44)	7.6 (1.1)	4.1 [2.01-6.89]
	2	38-56	H1_hu_N2	0.19 [0.14-0.25]	5.0 (1.4)	10.4 (2.5)	5.9 [4.23-7.96]

^a^Duration associated with Ct > 35 (M gene RT-PCR).

^b^Duration associated with Ct ≤ 35 (M gene RT-PCR ).

^c^Reproduction ratio: number of secondary infections caused by an infectious pig during its entire infectious period.

**Table 3:** Factors associated with the durations of latency and infectiousness in SIV infected piglets.

		**Latency (days)**		**Infectiousness (days)**	
**Variables and categories**	***n***	**Mean (sd)**	***P*value (F test)**	**Mean (sd)**	***P*value (F test)**
**Gender**			0.6		0.47
M	56	2.3 (1.4)		7.0 (2.5)	
F	51	2.1 (1.2)		7.3 (2.2)	
**Farm**			< 0.001		< 0.001
#A	34	2.2^b^ (1.0)		6.8^a^ (2.6)	
#B	31	1.4^a^ (0.4)		5.9^a^ (1.5)	
#C	42	2.7^b^ (1.7)		8.3^b^ (2.1)	
**Age at SIV infection time (days)**			< 0.001		0.004
≤ 50	61	2.6^a^ (1.5)		7.8^b^ (2.4)	
]50 – 80]	24	2.0^ab^ (1.0)		6.4^a^ (2.5)	
> 80	22	1.4^b^ (0.4)		6.2^a^ (1.6)	
**Mean HI titre (log2) of the piglet (7 days of age)**			0.44		0.19
Low (≤5.9)	24	2.5 (1.8)		7.8 (2.0)	
Moderate (]5.9-6.9])	34	2.0 (0.97)		7.3 (2.0)	
High (>6.9)	49	2.2 (1.3)		6.7 (2.7)	
**H1**_**av**_**N1 HI titre (log2) of the piglet (7 days of age)**			0.005		0.07
Low (≤5.3)	26	2.8^a^ (1.6)		8.0 (2.1)	
Moderate (]5.3-7.3])	24	2.4^ab^ (1.6)		7.2 (2.3)	
High (>7.3)	57	1.8^b^ (0.9)		6.7 (2.4)	
**H1**_**hu**_**N2 HI titre (log2) of the piglet (7 days of age)**			0.23		0.99
Low (≤5.3)	24	2.1 (1.7)		7.2 (1.9)	
Moderate (5.3-7.3)	27	1.9 (0.9)		7.1 (2.8)	
High (≥7.3)	56	2.4 (1.3)		7.1 (2.4)	
**Mean HI titre (log2) of the dam (7 days post-farrowing)**			0.20		0.005
Low (≤5.9)	48	2.3 (1.4)		7.8^a^ (1.6)	
Moderate (]5.9-6.9])	25	2.4 (1.4)		7.2^ab^ (2.5)	
High (>6.9)	34	1.9 (1.0)		6.1^b^ (2.3)	
**H1**_**av**_**N1 HI titre (log2) of the dam (7 days post-farrowing)**			0.07		< 0.001
Low (≤5.3)	43	2.5 (1.4)		7.8^a^ (2.1)	
Moderate (]5.3-7.3])	22	2.3 (1.7)		7.9^a^ (2.3)	
High (>7.3)	42	1.8 (0.9)		6.1^b^ (2.3)	
**H1**_**hu**_**N2 HI titre (log2) of the dam (7 days post-farrowing)**			0.38		0.71
Low (≤5.3)	36	2.1 (1.5)		7.4 (1.8)	
Moderate (]5.3-7.3])	32	2.5 (1.3)		7.1 (2.4)	
High (>7.3)	39	2.1 (1.2)		6.9 (2.7)	
**Dam parity**			0.02		< 0.001
0–1	23	1.8 (0.9)		7.1^a^ (1.8)	
2–3	55	2.5 (1.5)		8.0^a^ (2.3)	
≥4	29	1.8 (1.0)		5.6^b^ (2.2)	
**Number of stillborn or mummified piglets**			0.23		0.62
0 or 1	55	2.0 (0.9)		7.0 (2.2)	
2 or more	52	2.3 (1.6)		7.3 (2.5)	
**Number of cross-fostered piglets in the litter**			0.001		0.28
None	39	1.9^a^ (0.9)		6.9 (2.0)	
Between 1 and 4	44	2.0^a^ (1.1)		7.0 (2.4)	
More than 4	24	3.0^b^ (1.8)		7.8 (2.8)	
**H1**_**hu**_**N2 specific seroconversion**			0.69		0.799
yes	29	2.1 (1.4)		7.2 (2.4)	
no	78	2.2 (1.3)		7.1 (2.4)	

Means with different upper script letters (^a^,^b^) between different categories of a single variable are significantly different (*P* < 0.05, Tukey multiple comparison post-hoc test).

### Characteristics associated with age at first shedding and at seroconversion

The serological statuses of the dam one week after farrowing and of the 1 week-old piglet were highly correlated (R2 = 0.86, *P* < 0.001). One of the two variables was therefore retained in the final regression model (selection based on model quality). According to the multivariate Cox regression model, piglets which were the first SIV shedders and which initiated the observed outbreaks were more likely to be born to dams that received a large number of cross-fostered piglets (> 4) and which had low HI titres one week after farrowing (resulting in low HI titres in piglets at 1 week of age). These piglets were also from litters in which more than 2 mummified or stillborn piglets were observed at farrowing (Table 4).

**Table 4:** Final model for characteristics of piglets associated with time to swine influenza virus shedding.

**Variables and categories**	**Hazard ratio**	**Confidence interval (95%)**	***P*value**
**Mean HI titre (log2) of the dam**			< 0.001
**7 days after farrowing**			
Low (≤ 5.9)	2.4	1.8 – 3.3	
Moderate (]5.9-6.9])	1.6	1.2 – 2.3	
High (> 6.9)	-	-	
**Number of cross-fostered piglets in the litter**			< 0.001
None	-	-	
Between 1 and 4	0.98	0.77 – 1.3	
More than 4	3.7	2.5 – 5.4	
**Number of stillborn or mummified piglets**			0.01
0 or 1	-	-	
2 or more	1.4	1.1 – 1.7	

Cox proportional hazard model, *n* = 346 piglets, 304 events.

Characteristics related to seroconversion events were only evaluated for the H1_hu_N2 subtype as seroconversions to H1_av_N1 were rarely observed. Piglets that seroconverted post-H1_hu_N2 infection were more likely to be infected after 80 days of age and born to dams with a low H1_hu_N2 HI titre one week after farrowing (resulting in low HI titres at 1 week of age in piglets) (Table 5). Hence, censored piglets (no seroconversion observed before slaughter) were mainly born to sows with high HI titres and infected in early life when they still had passive immunity.

**Table 5:** **Final model for characteristics of piglets associated with seroconversion directed towards H1**_**hu**_**N2.**

**Variables and categories**	**Hazard ratio**	**Confidence interval (95%)**	***P*value**
**H1**_**hu**_**N2 HI titre of the dam**			< 0.001
**(7 days after farrowing)**			
Low (≤ 5.3)	5.1	3.1 – 8.5	
Moderate (]5.3-7.3])	2.3	1.3 – 4.1	
High (> 7.3)	-	-	
**Age at infection time (days)**			< 0.001
No detected infection	-	-	
≤ 50	0.2	0.1 – 0.5	
]50 – 80]	1.1	0.6 – 1.9	
> 80	4.6	2.6 – 8.2	

Cox proportional hazard model, *n* = 346 piglets, 140 events.

## Discussion

The follow-up of individual piglets in farms affected by recurrent influenza-like outbreaks demonstrated the ability of SIVs to persist in an enzootic form within a farrow-to-finish pig population. Our investigations confirmed the occurrence of outbreaks of SIV etiology, affecting all batches within a farm, and sometimes with repetitions in the same batch. A recurrent SIV infection occurring systematically in nursery around 50 days of age was apparent in farms #A and #C. This epidemiological form of influenza infection could also be encountered during the fattening phase, with several consecutive SIV passages in the same pigs, as shown by Farm#B. Multiple infections by viruses of H1_av_N1 and H1_hu_N2 subtypes were detected consecutively or even sometimes simultaneously in the same animal, in all 3 farms. Reassortant viruses were also isolated confirming that co-infections by different virus subtypes, facilitated in these recurrent infections, are propitious to reassortment events [33]. From our results, the other infectious agents investigated did not seem to be associated with SIV infection recurrence but could explain differences in disease severity, such as the PRRSV co-infection in Farm#B batch #3. It was suggested from the clinical and lesion data that these recurrent influenza outbreaks affected pig performances and could be an important aggravating factor for the Porcine Respiratory Disease Complex (PRDC) [6,7].

Serological data highlighted the absence of seroconversion in animals infected early, when maternal antibodies were present. HI tests using H1N1 and H1N2 viruses isolated in Farm#A, batch#1, as antigens were performed on all sera taken from 10 pigs selected in this batch, as well as ELISA tests. They confirmed that the humoral response in these piglets was impaired (data not shown). The absence of full protection by SIV maternally derived antibodies (MDA) and an impaired humoral response following an infection occurring in this context, have been described previously [34,35]. Thus, when piglets having maternally derived antibodies were inoculated with an H1N1 virus, they were protected against the clinical consequences of the flu infection, but developed a weaker immunity than piglets infected without MDA [34]. The formation of anti-HA antibodies was almost suppressed and the T-cell response was also weaker. In the same study, it was observed that infected piglets with MDA shed virus for a longer time, in agreement with our observations in piglets infected before 50 days of age. It can also be noted that the negative impact of MDA on piglet’s immune responses was also reported post-vaccination against influenza [36,37].

Piglets that seroconverted against the H1_hu_N2 subtype generally had low antibody titres at 1 week of age (because they were born to sows with low antibody titres) and were infected after 80 days of age. This specific seroconversion, which occurred mainly in piglets from Farm#B, did not protect them from subsequent infection by another virus subtype, i.e. H1_av_N1. This latter boosted the humoral anti-haemagglutinin response against the first infecting virus subtype while the specific response against the second subtype was weak. Even if some partial heterosubtypic immunity between H1N1 and H3N2 subtypes has been described [38], clinical protection was only observed experimentally after subsequent infections with H1N2 and H1N1 virus subtypes [39,40]. Moreover, the secondary infection was found to enhance the serological response against the primary subtype, similarly to our observations.

In this context of recurrent influenza outbreaks, the propagation potential of the SIV infections examined was high (*R* values between 2.5 and 6.9) with shedding durations of more than 7 days at the individual-level, resulting in a total outbreak duration of up to 1 month at the population level. Few data for *R* estimation of influenza in swine populations are available. One estimate (*R*_*0*_ = 2) based on literature data was used to model the transmission dynamics within a confined animal operation and the risk of transmission to humans [41]. Although our *R*-estimates are much larger, they are consistent with data from recent experimental transmission trials with an American triple reassortant H1N1 virus [42,43]. The high *R* values obtained in the present study suggest that an infected pig can infect between 3 to 7 pigs, on average, during its infectious period with an inflow of new susceptible pigs. Larger R estimates might be expected especially in fattening pigs without remaining maternal antibodies as several outbreaks in Farm#B resulted in 100% positive animals at the first sampling time. This theoretical estimation implies that the probability of virus maintenance within the population is extremely high. These high *R*-estimates could be explained by two different phenomena depending on the time of initiation of the infection sequence in a group of animals. Indeed, in a population with no remaining MDA (age greater than 80 days), large *R* values were related to high attack rates (*r*). In contrast, younger animals (below 50 days of age) exhibited lower attack rates but much longer shedding periods. A recent experiment showed that transmission was significantly reduced in the presence of MDA homologous to the strain used for challenge [43]. Only 1/20 sentinel pig in this group was infected whereas all pigs in groups with heterologous maternal immunity or with no MDA were infected, resulting in *R*_*0*_ estimates close to our largest estimate [43]. Our results indicate that the *R* estimates were always significantly greater than 1, even in the presence of MDA, which suggests that vaccine strains are not close enough to the field circulating strains to prevent transmission. In the specific case of a pig farm, the population cannot be considered as a homogeneous population as the individuals are segregated in different subpopulations corresponding to different batches of different ages. Thus, viral persistence from one batch to another would suggest poor internal biosecurity between batches, leading to a situation close to population homogeneity. These infectious outbreaks were mainly initiated by piglets born to sows delivering weak maternal immunity, from litters with numerous cross-fostered piglets and with an abnormally large number of mummified and stillborn piglets. The numerous mummified and stillborn piglets in these litters would suggest an infectious event in the dam shortly before farrowing. This is supported by serological profiles of the dams that led to hypothesize an active circulation of SIVs in the reproductive herd despite vaccination, with an increasing probability of exposure linked to age. Even if no SIV could be isolated from these sows, such infections occurring during late gestation and leading to mummified and stillborn piglets, cannot be excluded. To the best of our knowledge such SIV infections in sows leading to contamination of the offspring after birth have never been shown. If it was verified, it would also suggest early events in the infection process, involving the infection of susceptible piglets as early as the lactation phase and amplified by movements of piglets (cross-fostering, mingling at weaning). Other piglets become infectious with the waning of maternal antibodies. Such piglets infected in the presence of maternal antibodies have an impaired immune response and hence remain susceptible to another SIV infection. Further work is needed to fully understand the mechanisms involved in the impairment of the immune response in relation to age at infection time and/or presence of residual maternal antibodies. However, it can already be emphasized that all these conditions result in an infectious process sometimes lasting more than 30 days at the batch-level, which is enough for a new susceptible batch to be exposed to piglets still infectious from the previous one. Those results were obtained from detailed individual investigations from only 3 farms. Generalization of results especially mechanisms strictly related to farm characteristics should be considered with care. The results are also specific to the epidemiological situation of the area where only H1_av_N1 and H1_hu_N2 subtypes are currently circulating. Different patterns might be observed in other areas with other circulating SIVs. However some assumptions on mechanisms involved in the within-farm maintenance of SIVs can be suggested.

Factors jointly affecting the recurrence of SIV infection at the farm-level include the absence of a break in the infectious cycle (due to the short period of time between subsequent groups of contemporary pigs with susceptible and infectious animals sharing the same premises), the existence of subpopulations of piglets with an impaired immune response depending on their age and/or the presence of remaining maternal antibodies, and co-infections by different virus subtypes with high spreading potential. Management of this infectious process requires the identification of subpopulations and appropriate management of these subgroups within farms, the main focus being to limit mingling practices. Shortage of the infectious cycles between batches also requires reinforcement of internal biosecurity and strict age-segregated rearing with an all-in/all-out policy at the compartment level.

## Competing interests

The authors declare that they have no competing interests.

## Authors’ contributions

NR conceived and coordinated the study, participated in the follow-up study, analyzed the data and drafted the manuscript. SH coordinated the laboratory work (RT-PCR, HI tests, ELISA), analyzed the laboratory data and participated in their interpretation. EE, VD and FE organized and participated in the follow-up study in the 3 farms. NB performed the virological and serological analyses. MA participated in data analysis and transmission parameters estimation. CC participated in the follow-up study and in performing serological analyses. FM participated in the study coordination. GS supervised the laboratory work, interpreted the data and helped draft the manuscript. All co-authors revised the manuscript and approved the final submitted version. All authors read and approved the final manuscript.

## Supplementary Material

Additional file 1 **Estimation of individual Latency and Infectiousness durations from individual virological data.** Example of a polynomial (2^nd^ order) regression on SIV virological individual data (Ct values). Solutions corresponding to Ct =35 and Ct = 45 are drawn out to estimate the time interval between 35 < Ct ≤ 45 (latency period) and the time when Ct ≤ 35 (shedding period). Click here for file
