# Supplementary figures and images for: Dynamics of influenza A virus infections in permanently infected pig farms: evidence of recurrent infections, circulation of several swine influenza viruses and reassortment events
Source: Vet Res. 2013 Sep 4;44(1):72. doi: 10.1186/1297-9716-44-72 (PMC3846378; doi:10.1186/1297-9716-44-72)

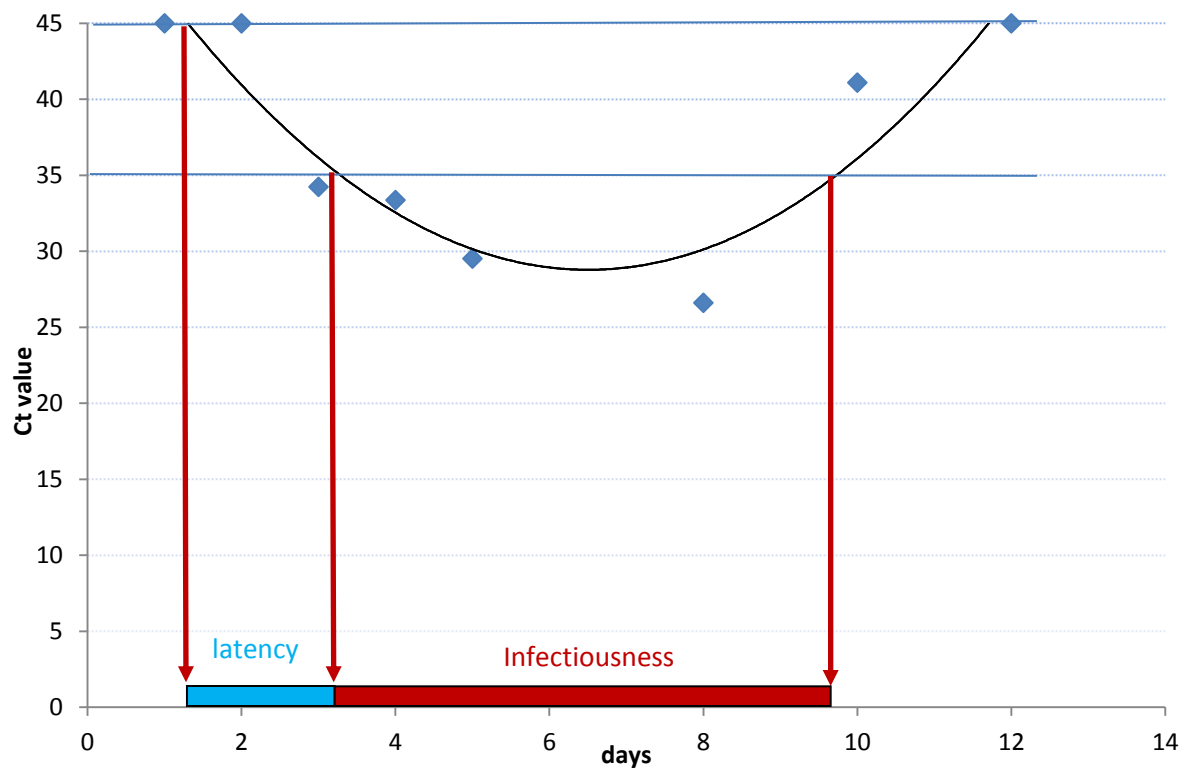

Supplement: Additional file 1 — Estimation of individual Latency and Infectiousness durations from individual virological data. Example of a polynomial (2nd order) regression on SIV virological individual data (Ct values). Solutions corresponding to Ct =35 and Ct = 45 are drawn out to estimate the time interval between 35 < Ct ≤ 45 (latency period) and the time when Ct ≤ 35 (shedding period). [file 1297-9716-44-72-S1.pdf]
